# Paul Epstein: 1943-2011

**DOI:** 10.1289/ehp.120-a12

**Published:** 2012-01-01

Paul Epstein succumbed to non-Hodgkin’s lymphoma on Sunday, 13 November 2011, 3 days before his 68th birthday. He began his career as a physician working in a low-income community in Boston, Massachusetts. In 1978–1980 he and his wife Andy, a nurse, went to a newly independent Mozambique, where he served as a volunteer chief of medicine at the Central Hospital in Beira. After returning to Boston, he continued to practice family medicine, and he also enrolled in a master’s program in tropical public health at Harvard University. During that time, he worked in an East Cambridge health center and began to describe the connections between outbreaks or reemergence of infectious diseases in response to climate change.

In 1992 he attended the UN Conference on Environment and Development (known as the “Earth Summit”) in Rio de Janeiro, Brazil, which focused on the environmental impacts of poverty and the waste of natural resources. He was inspired by this event, and later became one of the few physicians at the center of the evolving science of climate change and its effects on human health. He joined with colleagues Dick Levins, Howard Hu, and others at Harvard School of Public Health to form the New Diseases Group, which led to several publications and a popular course on global change and public health. Eventually, he became associate director of the Center for Health and the Global Environment, where he spent 15 years expanding knowledge and developing solutions to the emerging critical condition of the planet. He contributed to several articles published in *Environmental Health Perspectives* (*EHP*), served as a member of *EHP* ’s Editorial Review Board, and wrote a guest editorial in 2007 [Epstein PR. Climate Change: Healthy Solutions. Environ Health Perspect 115:A180].

In recent years, Epstein continued to speak out, collaborate with researchers, and teach about the public health consequences of the unhealthy energy choices we have made, as well as the urgent need to seek alternatives to fossil fuel as a primary source. He documented numerous examples in both developing and wealthy countries of human health impacts of extreme weather events and shifting climates. *Changing Planet, Changing Health*, a book cowritten with Dan Ferber and released in April 2011, summarizes his lifetime of work as a scientist, physician, leader, and visionary thinker. One of his most recent media appearances, earlier this year, was captured in a Greenpeace memorial tribute (http://www.greenpeace.org/usa/en/news-and-blogs/campaign-blog/in-memoriam-dr-paul-epstein-1944-2011-thank-y/blog/37841/).

He led a varied and productive life in clincal and public health over four decades. The world is a better place because of Paul Epstein’s work and the young people he inspired to continue this work.

